# Chemogenetic and Optogenetic Manipulations of Microglia in Chronic Pain

**DOI:** 10.1007/s12264-022-00937-3

**Published:** 2022-08-17

**Authors:** Sebastian Parusel, Min-Hee Yi, Christine L. Hunt, Long-Jun Wu

**Affiliations:** 1 Department of Neurology, Mayo Clinic, Rochester, MN 55905 USA; 2 Faculty of Psychology and Neuroscience, Maastricht University, Maastricht, MD, 6200 The Netherlands; 3 Department of Pain Medicine, Mayo Clinic, Jacksonville, FL 32224 USA; 4 Department of Neuroscience, Mayo Clinic, Jacksonville, FL 32224 USA; 5 Department of Immunology, Mayo Clinic, Rochester, MN 55905 USA

**Keywords:** Chronic pain, Microglia, Optogenetics, Chemogenetics, DREADDs, ReaChR

## Abstract

Chronic pain relief remains an unmet medical need. Current research points to a substantial contribution of glia-neuron interaction in its pathogenesis. Particularly, microglia play a crucial role in the development of chronic pain. To better understand the microglial contribution to chronic pain, specific regional and temporal manipulations of microglia are necessary. Recently, two new approaches have emerged that meet these demands. Chemogenetic tools allow the expression of designer receptors exclusively activated by designer drugs (DREADDs) specifically in microglia. Similarly, optogenetic tools allow for microglial manipulation *via* the activation of artificially expressed, light-sensitive proteins. Chemo- and optogenetic manipulations of microglia *in vivo* are powerful in interrogating microglial function in chronic pain. This review summarizes these emerging tools in studying the role of microglia in chronic pain and highlights their potential applications in microglia-related neurological disorders.

## Introduction

Neuropathic pain is a chronic condition that results in pain hypersensitivity and allodynia (pain responses to normally innocuous stimuli) after nerve damage that can occur after a host of insults, such as physical injury, diabetes, or autoimmune diseases [1]. When tissue damage has healed, however, neuropathic pain does not resolve [2]. A growing body of evidence indicates that microglia, as central nervous system (CNS) resident immune cells, play an important role in the pathogenesis of neuropathic pain [3–5]. Indeed, specific ablation or inhibition of microglia prevents the development of neuropathic pain [6–8]. In addition, recent progress highlights intimate microglia-neuron interactions in chronic pain [5, 9].

Microglia undergo functional changes during chronic pain states. In homeostatic conditions, microglia dynamically respond to changes in the microenvironment with their remarkably motile processes [10–13]. However, in response to peripheral nerve injury, microglia become activated and promote chronic pain. A major known mechanism for microglia to contribute to this process is through the release of cytokines and other mediators, such as interleukin-1 beta (IL-1β), IL-6, tumor necrosis factor alpha (TNFα), prostaglandin E_2_, brain-derived neurotrophic factor, and reactive oxygen species. These signals can lead to chronic pain [5, 14]. In addition to diffusible molecules, microglia also contribute to chronic pain hypersensitivity by adopting new functional roles, such as altered transcriptional activation and phagocytosis [9]. However, it is important to note that microglial activation during chronic pain states is not always detrimental because they are indeed heterogeneous [15, 16]. Recent findings identified a subpopulation of activated microglia playing a beneficial role in resolving chronic pain after peripheral nerve injury [17].

The cellular mechanisms of microglia in chronic pain have been investigated through pharmacological approaches. For example, systemic inhibition of microglia and macrophages by the broad inhibitor minocycline attenuates pain hypersensitivity [7, 8]. However, minocycline also has inhibitory effects on other cells, such as neurons, astrocytes, and T-cells [18–20]. Inhibitors of microglia through the colony-stimulating factor 1 receptor (CSF-1R), such as PLX5622 [21, 22] and neutralizing colony-stimulating factor 1 (CSF-1) antibody, also reduce microglial activation and proliferation in the spinal dorsal horn after nerve injury and alter pain responses [21, 23]. Specifically, mechanical allodynia and thermal hyperalgesia are attenuated in CSF-1 inhibitor-treated mice with chronic pain [21, 23–26]. However, CSF-1R inhibition induces off-target effects in other peripheral immune cells expressing the receptor [22, 27].

The importance of spinal microglia in the development of chronic pain has also been demonstrated by using genetic approaches to remove key microglial genes such as P2X4 [28], P2X7 [29], CX3CR1 [30], TRPM2 [31], P2Y12 [32], and Hv1 [33]. In addition, multiple Cre lines including CX3CR1 [34], Sal1 [35], TMEM119 [36], HexB [37], and P2Y12 [38] have been developed to target microglia. However, the development of advanced tools that provide temporal accuracy and spatial specificity is still needed. In the past few years, precise and selective methods for manipulating microglia have been used to study their involvement in chronic pain. Here, we introduce recent advances in how microglia control the pathophysiology of pain by using chemogenetic and optogenetic approaches.

## Chemogenetic Approaches in Microglia

**Fig. 1:**
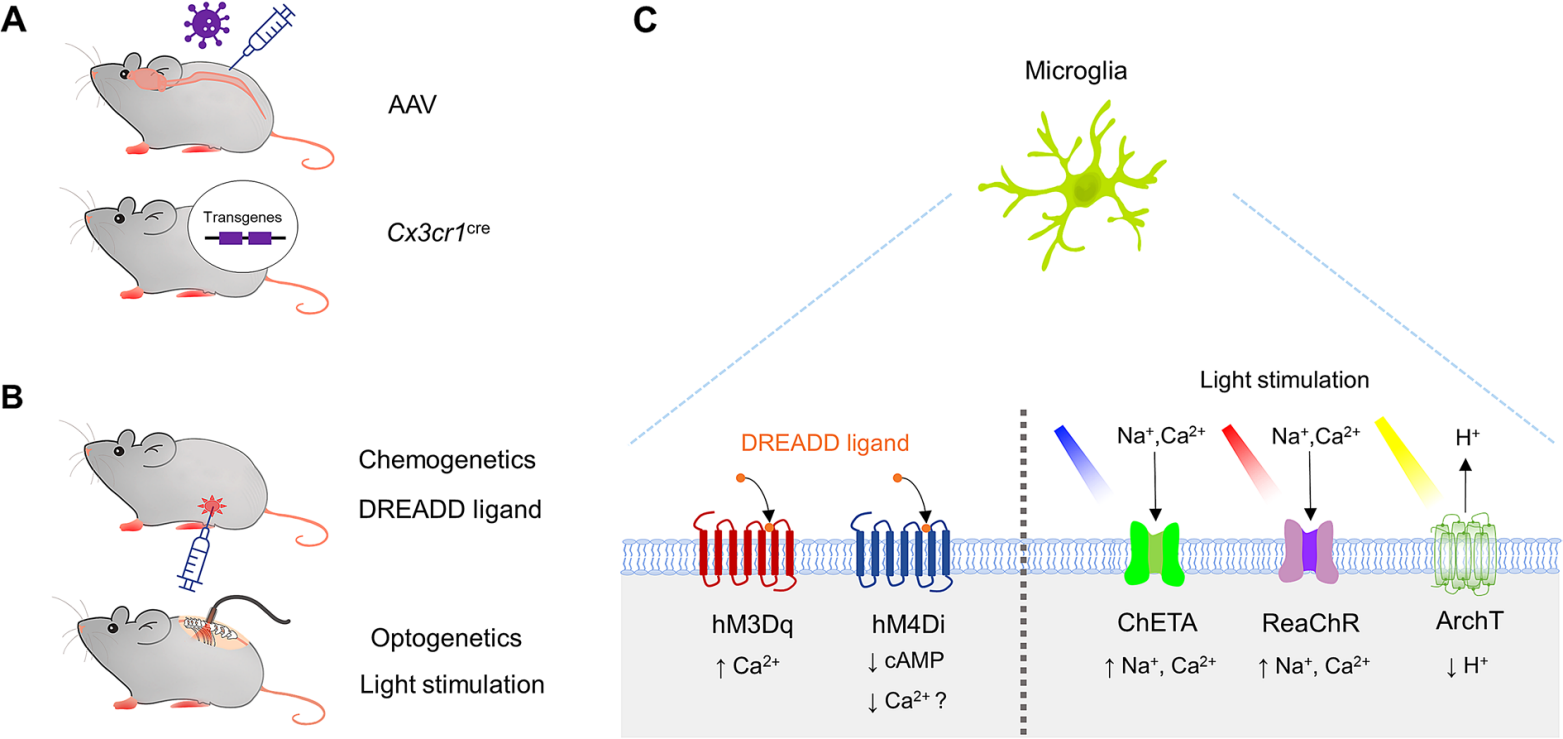
Chemogenetic and optogenetic approaches in microglia. **A** Viral-vector-mediated (e.g. AVV) or gene knock-in approaches (e.g. *Cx3cr1*^cre^) introduce DREADDs or opsins specifically in microglia. **B** Activation of DREADDs (chemogenetics) or opsins (optogenetics) in microglia by a DREADD ligand (e.g. CNO) or specific light stimulation. **C** In microglia, a DREADD ligand binds to hM3Dq or hM4Di, to activate Gq- or Gi-coupled signaling; light stimulation of opsins opens non-selective cation channels (such as ReaChR or ChETA) or proton pumps (such as ArchT)

Chemogenetic approaches refer to the expression and activation of DREADDs [39, 40]. DREADDs allow the selective interrogation of multiple G-protein-coupled receptor (GPCR) signaling cascades, including Gq, Gi, and Gs in various cell types [40]. DREADDs can be specifically activated in a cell type of choice by locally or systemically applying a specific ligand, such as clozapine N-oxide (CNO), with minimal off-target effects. Chemogenetic approaches have historically been used in neurons to interrogate the neuronal circuitry underlying behaviors [41–43]. Similarly, numerous studies have also applied DREADD approaches in astrocytes to investigate their physiological alterations in GPCR-mediated Ca^2+^ signaling [44], memory [45–47], neuroinflammation [48], and pain [49, 50]. Microglia express a number of GPCRs that are important for various microglial functions [51]. In particular, the microglial signature P2Y12 receptor is a Gi-coupled GPCR involved in the chemotaxis of processes towards ATP/ADP, which can occur after injury [52] and during the development of neuropathic pain [32, 53, 54]. Of the available chemogenetic GPCRs, Gi- (e.g. hM4Di) and Gq-signaling (e.g. hM3Dq) DREADDs have been used to investigate the functions of microglia in the CNS (Fig. 1).

### Chemogenetic Manipulation of CNS Microglia

Several studies have used chemogenetic approaches in microglia (Table 1). The Watkins’s lab was the first to use Gi and Gq DREADDs in rat microglia by viral expression [55, 56]. Spinal microglia were transfected with adeno-associated virus (AAV) 9 containing DREADDs driven under the CD68 promoter. AAVs have been successfully used to target various cell types in the CNS. However, microglial transduction *in vivo* is complicated, and it may not achieve robust transfection levels [57]. However, in the Watkins studies, microglial DREADD transfection by AAV led to functional DREADD expression in the spinal cord. Microglial Gi DREADD activation can attenuate pro-inflammatory signaling including through the nuclear factor of the kappa light polypeptide gene enhancer in B-cell inhibitor alpha, NLR family pyrin domain-containing 3 (NLRP3), and IL-1β [55]. On the other hand, Gq DREADDs mediate microglial activation and cytokine release [58, 59], potentially through the mobilization of intracellular Ca^2+^. Thus, Gq DREADD activation induces pro-inflammatory mediator production, while Gi DREADD activation inhibits lipopolysaccharide- (LPS) and chemokine (C-C motif) ligand 2-induced inflammatory signaling *in vitro* [56]. One potential confounder in these studies is the possibility that microglia might react with an immune response to AAV transfection [57]. However, it has been reported that AAV vectors (in contrast to adenovirus-based vectors) cause minimal immune reactions [60]. Nonetheless, careful use of adequate controls (DREADD expression without DREADD ligand administration) is essential to distinguish the effects of chemogenetic manipulation from the side effects of immune reaction to viral infection.

**Table 1:** Chemogenetic applications in microglia

References	DREADD expression	DREADD type	DREADD ligand administration (CNO)	Effects
Grace *et al.* [55]	hM4Di DREADDs *via* CD68-driven AAV9 (Spinal cord)	Gi	20 μg/h i.t. for 5.5 d	Prevention of morphine-induced central sensitization
			20 μg/h i.t. for 7 d	Attenuation of pro-inflammatory signaling
Grace *et al.* [56]	hM4Di DREADDs *via* CD68-driven AAV9 (Spinal cord)	Gi	1 mg/kg i.p. or 60 μg i.t.	Inhibition of pro-inflammatory signaling and reversal of neuropathic pain
	hM3Dq DREADDs *via* CD68-driven AAV9 (Spinal cord)	Gq	1 mg/kg i.p. or 60 μg i.t.	Induction of pro-inflammatory signaling and induction of chronic pain
Binning *et al.* [65]	*Cx3cr1*^creER/+^:*R26*^LSL-hM3Dq/+^	Gq	1 mg/kg i.p.	Increase in phagocytic activity
			1 mg/kg i.p. daily for 4 d	Attenuation of LPS-induced pro-inflammatory signaling
Saika *et al.* [61]	*Cx3cr1*^cre/+^:*R26*^LSL-hM4Di/+^	Gi	10 mg/kg i.p. or 20 nmol i.t.	Attenuation of neuropathic pain after PSL
Saika *et al.* [62]	*Cx3cr1*^cre/+^:*R26*^LSL-hM3Dq/+^	Gq	1 mg/kg i.p. or 2 nmol i.t.	Induction of chronic pain
Yi *et al.* [63]	*Cx3cr1*^creER/+^:*R26*^LSL-hM4Di/+^	Gi	5 mg/kg i.p. daily for 3 d	Delayed development of neuropathic pain (DREADD activation 3 d prior to SNT) Attenuation of neuropathic pain (DREADD activation 3 d after SNT)
Klawonn *et al.* [66]	*Cx3cr1*^creER/+^:*R26*^LSL-hM4Di/+^	Gi	2 mg/kg i.p.	Prevention of LPS-induced place aversion
	hM3Dq DREADDs *via* Cre-inducible lentivirus (Dorsal striatum)	Gq	2 mg/kg i,p,	Induction of place aversion
Császár *et al.* [67]	*Cx3cr1*^creER/+^:*R26*^LSL-hM3Dq- *CGaMP5g–tdTomato*/+^	Gq	0.5 mg/kg i.p. or 1 μg/kg i.p. (DCZ)	Increase in microglial intracellular Ca^2+^ Withdrawal of microglial processes around arterioles

CNO, clozapine N-oxide; AAV, adeno-associated virus; DREADD, designer-receptor-exclusively-activated-by-designer-drug; i.t., intrathecal; i.p., intraperitoneal; LPS, lipopolysaccharide; PSL, partial sciatic nerve ligation; SNT, spinal nerve transection; DCZ, deschloroclozapine

**Fig. 2:**
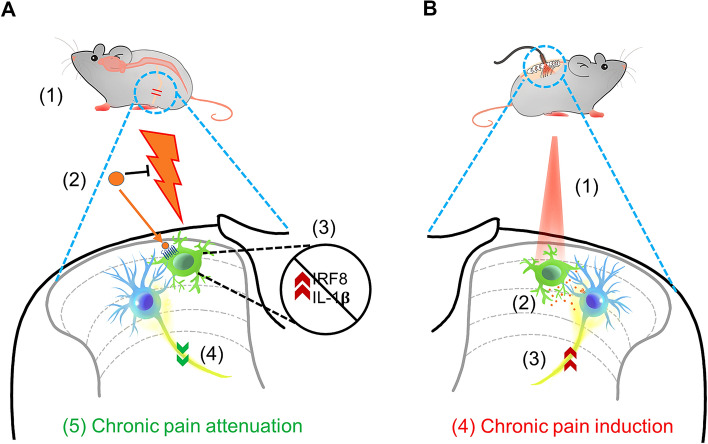
Mechanisms underlying chronic pain regulation by chemogenetic and optogenetic manipulation of microglia. **A** Chemogenetic activation of Gi DREADD attenuates neuropathic pain after peripheral nerve injury. After L4 spinal nerve transection (SNT) (1), CNO activation of Gi DREADD-expressing microglia (2) leads to microglial inhibition. Subsequently, SNT-induced microglial upregulation of IRF8 and IL-1β is inhibited (3). Thus, chemogenetic inhibition of microglia normalizes neuronal hyperactivity (4) and attenuates chronic pain behaviors after SNT (5). **B** Optogenetic activation of spinal microglia triggers chronic “microgliogenic” pain. Optogenetic stimulation of spinal microglia expressing ReaChR with red light (625 nm) (1) activates microglia and increases the Ca^2+^-dependent release of IL-1β (2), which sensitizes neuronal activity (3), leading to chronic pain behaviors (4)

Other studies have more commonly applied gene knock-in approaches to selectively express Gi/Gq DREADD in microglia [61–63]. The C-X3-C motif chemokine receptor 1 (CX3CR1) is highly expressed by microglia in the CNS and cells of mononuclear origin in the periphery [64]. The use of constitutive *Cx3cr1*^cre/+^:*R26*^LSL-hM4Di/+^ mice results in Gi DREADD expression in all CX3CR1-expressing cells including microglia and monocytes [61]. To exclusively express Gi DREADD in microglia but not peripheral monocytes, researchers have used inducible *Cx3cr1*^creER/+^:*R26*^LSL-hM4Di/+^ mice [63]. Due to the fact that blood CX3CR1^+^ cells have rapid turnover while microglia are longer-lived, it is possible to achieve greater microglial specificity by waiting for cell turnover after tamoxifen administration [34]. Indeed, DREADDs are expressed and co-localized only with Iba1^+^ microglia in the spinal cord and brain 4 weeks after tamoxifen injection [63]. Using these genetic knock-in mice expressing Gi/Gq DREADDs in microglia, further studies have interrogated their function and underlying mechanisms in chronic pain (Fig. 2) [61–63].

In addition to pain research, chemogenetic methods were also recently applied to microglia to investigate their functions in inflammation, vascular interaction, and aversive behaviors. For example, inducible *Cx3cr1*^creER/+^:*R26*^LSL-hM3Dq/+^ mice were used for the functional expression of microglial Gq DREADDs [65]. Gq DREADD activation induced intracellular Ca^2+^ elevation and the phagocytosis of FluoSpheres in primary microglia. Unexpectedly, chronic Gq DREADD activation attenuated the LPS-induced increase of pro-inflammatory cytokines, including TNFα, IL-1β, and IL-6 in the mouse brain. In line with these results, chronic Gq DREADD activation in microglia robustly increased social exploration 2 h after LPS-induced inflammation [65]. Recently, a Cre-inducible lentiviral vector has been used to express DREADDs in dorsal striatal microglia of *Cx3cr1*^creER/+^ mice [66]. Using this approach, both microglial Gq DREADD activation in naïve mice or LPS administration led to conditioned place aversion. Interestingly, microglial Gi DREADD activation before LPS administration prevents the development of conditioned place aversion without affecting markers of systemic inflammation [66]. Chemogenetic methods have been also applied to microglia to investigate neurovascular coupling changes [67]. *Cx3cr1*^creER/+^:*R26*^LSL-hM3Dq-*CGaMP5g–tdTomato*/+^ mice were used for the expression of microglial Gq DREADDs. Activation of Gq DREADDs in microglia that interact with arterioles and microvessels in the cortex led to the withdrawal of perivascular microglial processes around arterioles and reduced the cerebral blood flow in response to whisker stimulation. Taken together, these bi-directional chemogenetic approaches have proven to be powerful tools in interrogating microglial function in the brain.

### Chemogenetic Manipulation of Microglia in Chronic Pain

The first study applying chemogenetic approaches through the viral expression of DREADDs in microglia studied the contribution of microglia to morphine-induced persistent sensitization in rats [55]. While opiates have been regularly used for pain treatment, they paradoxically induce nociceptive sensitization known as opioid-induced hyperalgesia [68]. Exposure to opioids in healthy individuals has been demonstrated to lead to hyperalgesia in many clinical studies, although large-scale trials cannot be performed in human subjects for ethical reasons [69, 70] Morphine-induced persistent sensitization is associated with microglial inflammasome activation in the spinal cord [55]. Microglial Gi DREADD activation reduces pro-inflammatory signaling and prevents morphine-induced persistent sensitization. Similar to microglial Gi DREADD activation, pharmacological blockade of toll-like receptor 4, P2X7, or the inflammasome can all independently block morphine-induced sensitization [55]. Thus, the mechanism underlying the action of Gi DREADD in microglia may be related to Gi inhibition of Ca^2+^ elevation for pro-inflammatory cytokine production or release [58, 71].

Using viral expression of both Gq and Gi DREADD specifically in microglia, the group further studied the role of microglia in chronic pain in rats [56]. Gi DREADD activation in microglia rapidly reversed allodynia in neuropathic pain conditions. Mechanistically, microglial Gi DREADD activation attenuated the level of inflammatory mediators including nitric oxide (NO) and IL-1β. Microglial Gq DREADD activation, on the other hand, was able to induce allodynia in naïve male rats and increased the expression of pro-inflammatory mediators, such as NO, TNFα, Il-1β, and IL-6 [56].

After Cre-inducible DREADD mice were generated in 2016 [72], both Kiguchi’s group and our group investigated microglial function in chronic pain in mice selectively expressing DREADDs in microglia. Using constitutive *Cx3cr1*^cre/+^:*R26*^LSL-hM4Di/+^ mice, Kiguchi’s group showed that microglia expressing Gi DREADDs in the spinal cord, upon activation, alleviate pain sensitization after partial sciatic nerve ligation (PSL) [61]. Using inducible *Cx3cr1*^creER/+^:*R26*^LSL-hM4Di/+^ mice, we showed that Gi DREADDs are specifically expressed in microglia in adult mice [63]. Microglial Gi DREADD activation 3 days before L4 spinal nerve transection (SNT) delayed the development of allodynia while activation 3 days after SNT attenuated mechanical allodynia [63].

Several potential mechanisms underlying microglial Gi DREADD in pain attenuation have been proposed [63]. First, activation of microglial Gi DREADD signaling prevents microglial proliferation, known as the main source of microgliosis in neuropathic pain [4, 23]. Second, SNT-upregulated expression of interferon regulatory factor (IRF) 8, a transcription factor implicated in microglial transition to a reactive state [73], is prevented by Gi DREADD activation. Similarly, Il-1β as a critical mediator of neuropathic pain [73, 74], is also decreased by microglial Gi DREADD activation. Third, C-fiber-evoked field potentials *in vivo* are reduced upon activation of microglial Gi DREADD. These results complement and expand previous findings that selective microglial activation in the spinal cord promotes synaptic strengthening and synaptic plasticity between primary afferent C-fibers and spinal neurons [25, 75]. Together, these results indicate that Gi DREADD manipulation in microglia attenuates chronic pain by inhibiting microglial proliferation, neuroinflammation, and synaptic potentiation (Fig. 2A).

Microglial function in chronic pain was further investigated by using Gq DREADD in constitutive *Cx3cr1*^cre/+^:*R26*^LSL-hM3Dq/+^ mice [62]. After Gq DREADD activation, naïve male mice displayed allodynia and hyperalgesia. Further analysis showed that Gq DREADD activation led to a significant upregulation of inflammatory mediators (IL-1β, TNFα, CCL3, and CCL4) and microglial markers (Iba1, CD11b, IRF5, and IRF7). Importantly, these results point to a sex-specific mechanism, as both the behavioral correlates of pain and their potential underlying inflammation occurred in male but not female mice. After microglial ablation by PLX3397, an inhibitor of CSF1-R, Gq DREADD activation by CNO administration does not induce chronic pain hypersensitivity or the upregulation of inflammatory markers in male mice, further providing evidence that the chemogenetic activation of microglia is necessary for the development of chronic pain.

These findings are part of a larger literature finding an interesting sex-dependent microglial function in chronic pain. Although microglial activation (microgliosis) develops in both sexes in neuropathic pain models, emerging reports suggest that microglial inhibition resolves pain only in male mice. For example, microglia-targeted inhibitors (minocycline as well as inhibitors of TLR4, P2X4, or p38 mitogen-activated protein kinases) are effective in attenuating neuropathic pain in male but not female rodents [76–78]. One potential explanation is the involvement of T-cells in the development of chronic pain in females only, while males depend on microglia-related mechanisms [76]. In line with this idea, activation of Gi DREADD in CX3CR1^+^ cells attenuates mechanical allodynia after PSL only in male mice [61]. While Gq DREADD microglia can initiate sex-dependent differences in pain responses, we have recently found that microglial Gi DREADD activation attenuates neuropathic pain in both male and female mice after SNT [63]. This discrepancy might be explained by different genetic manipulations (constitutive *Cx3cr1*^cre/+^ *versus* inducible *Cx3cr1*^creER/+^ mouse lines impacting different sets of cell classes) or different CNO dose paradigms (one time-point only at 10 mg/kg *vs* three times at 5 mg/kg/day). Further, no sex differences have been reported in chronic pain attenuation after CX3CR1^+^ cells are genetically ablated [6]. Future studies are needed to understand these potential discrepancies and determine the circumstances under which microglia may engage in sex-dependent chronic pain responses.

## Optogenetic Approaches in Microglia

Optogenetics fuse genetic and optical procedures to allow the manipulation of specific cell populations, conferring the unique capability to sense and respond to light through light-sensitive proteins in behaving animals [79]. All known organisms express photon-sensitive receptor proteins, called rhodopsins. The main types of opsins found in microorganisms are bacteriorhodopsins and halorhodopsins, which are light-driven ion pumps/channels such as channelrhodopsins (ChRs), and sensors such as sensory rhodopsin [80]. Optogenetic approaches have been widely used to drive the depolarization or hyperpolarization of selected neurons in response to specific wavelengths of light, allowing scientists to interrogate complex circuits underlying behavior [81, 82] including pain sensation [83]. Recent advances have also enabled optogenetic approaches to be applied to glial cells such as astrocytes and microglia [84–87]. For example, optogenetic approaches have been used to dissect astrocyte functions in breathing [84], memory [45], and epilepsy [85]. Furthermore, a recent study used ChR2, a non-selective, depolarizing cation channel, to selectively activate astrocytes. Depolarized spinal astrocytes elicited chronic pain behaviors by inducing ATP release [86]. In the periphery, optogenetic activation of ChR2-expressing macrophages in the heart improves the electrical connections underlying conduction [88]. Here, we introduce how optogenetics has been applied to microglia to dissect their function in the CNS, with a particular focus on chronic pain (Fig. 1).

### Optogenetic Manipulation of CNS Microglia

Ionotropic signaling is an overlook mechanism underlying microglial interactions with the brain microenvironment [89]. Unlike neurons, microglia have few voltage-gated Na^+^ or Ca^2+^ channels *in vivo*. Microglia mediate ionic fluxes using multiple ion channels including K^+^ channels [90, 91], proton channels [92], transient receptor potential channels [93], pannexin-1 [94, 95], and purinergic ionotropic receptors [96]. The changes in microglial membrane potential in response to ion channel activation under pathological conditions are associated with the reactive microglial transition. For instance, prolonged increased K^+^ channel conductance often precedes the reactive state transition [51, 97]. K^+^ channels are also essential for microglial process surveillance and chemotactic responses to extracellular ATP/tissue injury [91, 98, 99]. However, it is not known whether the changes in membrane potential are either necessary or sufficient for microglial activation. Recent advances applying optogenetic approaches to microglia allow us to address these early questions [87, 100].

Only a few studies have used optogenetic approaches in microglia so far (Table 2). In a proof-of-concept study by Yamanaka’s group, ChR2 was expressed specifically in microglia by using transgenic *Iba1-tTA:tetOChR2(CS128S)-EYFP* mice [101]. Blue light stimulation depolarized microglia indicating its functional expression, but no further studies were conducted using these mice. For the first time, red-activated ChR (ReaChR) was expressed in microglia using *Cx3cr1*^creER/+^:*R26*^LSL-ReaChR/+^ mice [87]. The advantage of using ReaChR (a newer generation of non-selective cation channels) compared with ChR2 is its activation by red light, which has better penetration deeper into tissue with less light scatter than the blue/green light for ChR2 activation. In addition, the ReaChR current can be maintained with far less inactivation occurring during light stimulation [102]. Selective microglial ReaChR expression can be achieved using *Cx3cr1*^creER/+^:*R26*^LSL-ReaChR/+^ mice. In the spinal cord, rhodopsin protein is only co-localized with Iba1^+^ microglia [87]. ReaChR expression is also functional as red-light stimulation induces inward currents in spinal microglia, resulting in their depolarization. In addition, pro-inflammatory cytokines such as IL-1β are secreted by primary microglia after red-light stimulation and this requires extracellular Ca^2+^ influx [87]. Thus, optogenetic depolarization of spinal microglia is sufficient for Ca^2+^-dependent cytokine release.

**Table 2:** Optogenetic applications in microglia

References	Opsin Expression	Opsin Type	Light Stimulation	Effects
Tanaka *et al.* [101]	*Iba1-tTA:tetOChR2(CS128S)-EYFP*	ChR2	50 mW blue laser 500 ms pulses at 1 s intervals	Microglial depolarization
Yi *et al.* [87]	*Cx3cr1*^creER/+^:*R26*^LSL-ReaChR/+^	ReaChR	625 nm, red LED 45 ms light on, 5 ms light off, 20 Hz for 30 min	Microglial depolarization Increased microglial IL-1β expression Induction of chronic pain
Laprell *et al.* [100]	*Cx3cr1*^creER/+^:*R26*^LSL-ChETA-tdTomato/+^	ChETA	480 nm, blue LED 1 Hz light flashes for 20 min	Microglial depolarization Slowed chemotaxis response to laser burn
	*Cx3cr1*^creER/+^:*R26*^LSL-ArchT-EGFP/+^	ArchT	575 nm, yellow/green LED	Microglial hyperpolarization No effect on chemotaxis response to laser burn

ChR2, channelrhodopsin-2; ReaChR, red-activated channelrhodopsin; IL-1β, interleukin-1 beta ChETA a modified form of channelrhodopsin-2; ArchT, archaerhodopsin

Similarly, ChETA (a modified form of ChR2) was expressed in microglia using *Cx3cr1*^creER/+^:*R26*^LSL-ChETA-tdTomato/+^ mice [100]. ChETA activated by blue-light induced microglial depolarization and slowed the chemotaxis of processes in response to laser-induced tissue damage [100]. Microglia rapidly hyperpolarize when sensing ATP or neuronal hyperactivity [103]. Indeed, P2Y12-coupled K^+^ channel activation is part of the mechanism for rapid chemotactic reactions to laser injury or basal motility [91, 98, 99]. A slower chemotaxis response induced by optogenetic microglial depolarization indicates that ATP-mediated hyperpolarization is not only a concomitant phenomenon of microglial activation but is required for the rapid expansion of microglial processes towards injury. One caveat of using blue light stimulation is the potential off-target effects. For instance, a study showed that microglia alter inflammatory-related gene expression with different levels of blue light stimulation [104]. Nevertheless, the results using ChETA indicate a potential correlation between the membrane potential and the chemotaxis of microglial processes. The light-activated proton pump archaerhodopsin (ArchT) has also been expressed in microglia using *Cx3cr1*^creER/+^:*R26*^LSL-ArchT-EGFP/+^ mice [100]. Unlike ChR2 and its variants, ArchT activation by yellow/green light results in hyperpolarization. However, the ArchT-mediated hyperpolarization of microglia does not alter the electrophysiological responses of microglial to laser-induced tissue damage, nor does it affect chemotactic responses.

Microglia sense neuronal activity and the brain environment *via* Ca^2+^ signaling [59, 105]. Indeed, increased Ca^2+^ in microglia is strongly correlated with pathophysiological activation such as neuroinflammation [106], seizures [59], stroke [107], and neurodegeneration [108]. Since ChR2 and its derivatives are Ca^2+^-permeable ion channels [109], optogenetic activation of microglia allows for the direct manipulation of Ca^2+^ influx. As a result, ReaChR activation of microglia leads to Ca^2+^-independent cytokine release [87]. On the contrary, in Ca^2+^-free extracellular solutions, microglial chemotaxis to damage sites is significantly slowed, similar to the increased ionic influx during ChETA activation [100]. Therefore, these results suggest that optogenetic depolarization of microglia inhibits Ca^2+^ elevation, thus slowing the chemotaxis of microglial processes. The underlying mechanism might be due to the reduction of the driving force for Ca^2+^ during depolarization in microglia. Interestingly, previous studies found that the removal of extracellular Ca^2+^ alone induces the convergence of microglial processes, similar to that reported in seizures and stroke [110, 111]. Future experiments using *in vivo* Ca^2+^ imaging are needed to directly investigate whether optogenetic activation of microglia increases or decreases microglial Ca^2+^ signaling.

### Optogenetic Manipulation of Microglia in Chronic Pain

By using *Cx3cr1*^creER/+^:*R26*^LSL-ReaChR/+^ mice to exclusively express ReaChR in microglia, spinal microglia can be depolarized in real time to examine their function in pain behaviors (Fig. 2B). Red-light stimulation can be delivered locally to the lumbar spinal cord through optic fibers. After light stimulation (30 min at 20 Hz,) mechanical allodynia is evident one hour after stimulation and lasts for up to one week in mice [87]. These results are remarkable in that short-term optogenetic stimulation of spinal microglia alone induced long-lasting pain behaviors. The mere stimulation of spinal microglia through optogenetics in the absence of any inflammatory challenge, or nerve damage-elicited chronic pain [87, 112], suggests the intriguing possibility of “microgliogenic” pain that originates from microglial activation in the CNS.

Mechanistically, optogenetic stimulation of microglial ReaChR increases microglial proliferation, neuronal activity, and nociceptive transmission [87]. For example, C-fiber-evoked field potentials and neuronal C-fos expression in the dorsal horn are significantly increased after microglial optogenetic stimulation. Interestingly, IL-1β expression is increased 1–3 days after light stimulation of microglial ReaChR, which could be due to increased expression of NLRP3 inflammasome components and caspase-1. The IL-1 receptor antagonist IL-1ra is sufficient to prevent increased C-fiber-evoked field potentials by light stimulation and alleviate light-induced mechanical allodynia. Thus, optogenetic stimulation of spinal microglia triggers IL-1β release, which increases the neuronal activity underlying chronic pain behaviors (Fig. 2B). In sum, optogenetics allows specific and temporally-controlled manipulation of microglia to study their function in pain. This may provide additional benefit over chemogenetic approaches in that the optical stimulation has better spatial and temporal resolution.

## Conclusions and Outlook

Chemogenetics and optogenetics are two emerging approaches recently applied in the field of microglia research. DREADDs and opsin expression can be limited to microglia either by viral injection (e.g., AAV) or by promoter-driven conditional expression (e.g., CX3CR1). Unless activated, these proteins have no biological effects. Upon activation, existing DREADDs and opsins allow for a range of modulatory effects on microglia, including depolarization, hyperpolarization, and GPCR signal transduction. Chemo- and optogenetic manipulations of microglia are able to inhibit nerve injury-induced neuropathic pain or directly trigger chronic “microgliogenic” pain. However, it is important to note the limitations of chemo- and optogenetic approaches, as both use artificially-engineered proteins activated by designed stimuli. With chronic DREADD approaches, it has been suggested that repeated administration of CNO may lead to clozapine accumulation, which may have side effects unrelated to DREADDs [113]. The optogenetic stimulation of microglia might be unnaturally strong. In addition, it should be noted that effects may vary depending on the type of opsin and the frequency/intensity of light stimulation.

Here, we highlight the future of investigations of microglia by applying chemo- and optogenetic tools. (1) Microglia play a central role in many pathophysiological processes, such as in epilepsy [114], stroke [115], neurodegeneration [116, 117], depressive-like behaviors [118], memory deficits [119], and autoimmune neurology [120]. These new microglial tools will help illuminate the microglial mechanisms of neurological disorders. (2) Supraspinal microglial activation is also implicated in chronic pain [121–123]. Future studies will apply microglial tools to study their function in pain sensation, aversion, and comorbidities during chronic pain conditions. (3) Given the heterogeneity of microglia in neuropathic pain [15, 16] and their beneficial role in resolving chronic pain [17], it is unknown whether this heterogeneity also occurs when manipulating microglia using chemo- or optogenetic tools. Future studies will apply microglial tools to harness the beneficial function of alternatively-activated microglia during chronic pain conditions. (4) The ability of chemo- and optogenetic tools to directly manipulate Ca^2+^ levels allows the investigation of the role of Ca^2+^ signaling in microglia. Interrogation of the downstream Gi and Gq signaling in microglia is also made possible. Thus, the recent advances in microglial chemo- and optogenetic manipulations highlight the importance and novelty of these emerging tools in studying the function of microglia in neurological diseases, particularly in chronic pain.
